# Vascular Endothelial Growth Factor Increases during Blood-Brain Barrier-Enhanced Permeability Caused by *Phoneutria nigriventer* Spider Venom

**DOI:** 10.1155/2014/721968

**Published:** 2014-08-27

**Authors:** Monique C. P. Mendonça, Edilene S. Soares, Leila M. Stávale, Evanguedes Kalapothakis, Maria Alice Cruz-Höfling

**Affiliations:** ^1^Department of Pharmacology, Faculty of Medical Sciences, State University of Campinas (UNICAMP), 13084-971 Campinas, SP, Brazil; ^2^Department of Biochemistry and Tissue Biology, Institute of Biology, State University of Campinas (UNICAMP), 13083-970 Campinas, SP, Brazil; ^3^Department of General Biology, Institute of Biological Sciences, Federal University of Minas Gerais (UFMG), 31270-901 Belo Horizonte, MG, Brazil

## Abstract

*Phoneutria nigriventer* spider accidental envenomation provokes neurotoxic manifestations, which when critical, results in epileptic-like episodes. In rats, *P. nigriventer* venom (PNV) causes blood-brain barrier breakdown (BBBb). The PNV-induced excitotoxicity results from disturbances on Na^+^, K^+^ and Ca^2+^ channels and glutamate handling. The vascular endothelial growth factor (VEGF), beyond its angiogenic effect, also, interferes on synaptic physiology by affecting the same ion channels and protects neurons from excitotoxicity. However, it is unknown whether VEGF expression is altered following PNV envenomation. We found that adult and neonates rats injected with PNV showed immediate neurotoxic manifestations which paralleled with endothelial occludin, *β*-catenin, and laminin downregulation indicative of BBBb. In neonate rats, VEGF, VEGF mRNA, and Flt-1 receptors, glutamate decarboxylase, and calbindin-D28k increased in Purkinje neurons, while, in adult rats, the BBBb paralleled with VEGF mRNA, Flk-1, and calbindin-D28k increases and Flt-1 decreases. Statistically, the variable age had a role in such differences, which might be due to age-related unequal maturation of blood-brain barrier (BBB) and thus differential cross-signaling among components of the glial neurovascular unit. The concurrent increases in the VEGF/Flt-1/Flk-1 system in the cerebellar neuron cells and the BBBb following PNV exposure might imply a cytokine modulation of neuronal excitability consequent to homeostatic perturbations induced by ion channels-acting PNV neuropeptides. Whether such modulation represents neuroprotection needs further investigation.

## 1. Introduction

Accidents with venomous animals have been considered a neglected health public issue. Accidents caused by* Phoneutria nigriventer*, popularly known as the armed spider, are common in Southeast Brazil. The majority of them only cause pain, intense sudoresis, and local inflammation. However, accidents graded as severe (less than 0.5%) cause in addition cramps, tremors, blurred yellow vision, tachypnea, cardiovascular alterations, priapism, and convulsion and rarely death, those symptoms appearing to be more severe in children [1, 2].

Experimental studies in rats showed that the clinical symptoms of envenomation paralleled those of humans. Our studies have shown that the* Phoneutria nigriventer* venom induces blood-brain barrier breakdown through increased vesicular transcellular transport [3] followed by upregulation of the glycoprotein P (P-gp) efflux protein and by impairment of the paracellular pathway due to the displacement and phosphorylation of junctional proteins [4]. Apart from that, the venom induces vasogenic edema in hippocampus [5] and cytotoxic edema in rat cerebellum [6], reactive gliosis in the two regions [7], and neuronal activation (FOS+ induction) in regions associated with stress and motor activity [8]. In the cerebellum gray matter, mainly in the granular layer, PNV upregulates the water channel aquaporin-4 [9], a protein which regulates edema formation and resolution. All the changes have a pace and tend to be restored with time. The mechanism(s) underlying these effects is unknown, but hypoxia secondary to the respiratory and cardiovascular distress exhibited by animals could be a primary cause.

VEGF, an angiogenic cytokine which mediates vascular permeability [10], has been reported to protect neurons in adverse conditions by modulation of glutamatergic synaptic excitability and interference on Ca^2+^, K^+^, and voltage-gated Na^+^ channels physiology [11–13]. In hippocampus, the cytokine is considered an endogenous anti-convulsing factor to preserve pyramidal neurons from hyperexcitability [14]. VEGF exerts its action through the binding and activation of two transmembrane tyrosine kinase receptors: VEGFR-1 or Flt-1 [15] and VEGFR-2 or Flk-1 [16]. The receptors, in turn, modulate the VEGF signals that affect different cellular processes, so contributing for counteracting or aggravating the noxious stimuli.

In the light of the above, the current study aimed at determining whether the expression of VEGF, Flt-1, and Flk-1 and respective mRNAs of cerebellar neurons is altered in the course of BBB impairment caused by PNV in rats. Age-related alterations were investigated in neonate and young adult rats. We further analyzed the expression of glutamate decarboxylase (GAD), responsible for the synthesis of gamma-amino butyric acid (GABA) and of the calcium-binding protein calbindin-D28k (CaB), a marker of Purkinje cells. Such events were investigated because postnatal development continues for several months in the cerebellum and blood-brain barrier interface is immature in neonates. Besides, BBB of cerebellum has been already shown to be disrupted by PNV, and our hypothesis is that changes in the expression of VEGF/Flt-1/Flk-1 system may occur in parallel with BBB opening induced by ion channels-acting* P. nigriventer* venom and those changes could be age dependent.

## 2. Material and Methods

### 2.1. Ethics Statement

This study was approved by the Institution's Committee for Ethics in Animal Use (CEUA/IB/Unicamp, protocol 2403-1) and the experiments were carried out according to the Brazilian Society of Laboratory Animal Science (SBCAL) guidelines for animal use.

### 2.2. Venom

PNV was obtained by the electrical stimulation of spiders living in Arachnid Laboratory at the Department of Biology, IBS, UFMG, Belo Horizonte, MG, Brazil, and stored at −20°C until use.

### 2.3. Animals and Envenoming Procedure

Male Wistar rats (*Rattus norvegicus*) were obtained from an established colony maintained by the Central Animal House Service at Unicamp (CEMIB/Unicamp) and housed under standard laboratory conditions. Rats aged 14 days (P14 animals—neonate group) and 8–10 weeks (adult group) were used; they received a single intraperitoneal (i.p.) injection of PNV (1.7 mg/kg in 0.5 mL of 0.9% sterile saline solution), while to control groups the same volume of vehicle was given. PNV concentration was selected based on previous laboratory studies [9, 17]. Neonate rats were used for comparison with adult rats, since severe accidents by* Phoneutria* generally occur in children [1]. Time limits of 2, 5, and 24 hours corresponded to periods of peak of intoxication, beginning of clinical recovery, and no sign of intoxication at all, respectively [17].

### 2.4. Evaluation of Blood-Brain Barrier (BBB) Permeability

The integrity of the BBB was determined using Evans blue extravasation method. Briefly, 10 mL/kg of Evans blue (Vetec Química, Duque de Caxias, RJ, Brazil) dye solution (2% in 0.9% saline) was injected into the tail vein of adult rats (i.v.) at 2, 5, and 24 hours after i.p injection of 0.9% saline or PNV (1.7 mg/kg). Fifteen minutes after Evans blue injection, the rats were killed with an overdose of anesthetics and brain and cerebellum were rapidly removed and photographed.

### 2.5. Immunohistochemistry

At designated time intervals and after anesthesia, the animals (*n* = 4/time interval, *n* = 12 control, and *n* = 12 PNV-treated per age) were perfused transcardially with saline solution followed by 4% paraformaldehyde in 0.1 M PBS (phosphate buffered saline), pH 7.4. The cerebella were dissected out and were embedded in paraffin. Antibodies utilized were anti-VEGF (1 : 50, sc-7269), anti-Flt-1 (1 : 500, sc-316), and anti-Flk-1 (1 : 50, sc-315), all from Santa Cruz Biotechnology (Santa Cruz, CA, USA), CaB (1 : 1000, C2724, Sigma-Aldrich, St. Louis, MO, USA), and GAD (1 : 500, AB1511, Millipore, Billerica, MA, USA). Immunohistochemistry was performed in sequential coronal 5 *μ*m thick paraffin sections of the cerebellum as previously described [17]. To avoid procedure differences between control and envenomed groups, the immunohistochemistry for each protein (VEGF/Flt-1/Flk-1 and CaB/GAD) was performed concomitantly. Three images per region (molecular, granular, and Purkinje) of *n* = 4 rats, totaling 12 images per time/age/treatment, were captured using a BX51 microscope (Olympus Optical C. Ltd., Tokyo, Japan). Objective (20x), lens aperture, and light intensity were set for all images captured.

### 2.6. Western Blot (WB)

Standard Western blot analysis [4] of cerebellum lysates (*n* = 6/time interval, *n* = 18 control, and *n* = 18 PNV treated per age) was performed using rabbit polyclonal antibody against CaB (1 : 2000, C2724, Sigma-Aldrich, St. Louis, MO, USA), Flt-1 (1 : 500, sc-316), Flk-1 (1 : 250, sc-315), both from Santa Cruz Biotechnology (Santa Cruz, CA, USA), GAD (1 : 1000, AB1511, Millipore, Billerica, MA, USA) and laminin (1 : 500, L9393, Sigma-Aldrich, St. Louis, MO, USA), mouse monoclonal antibody against VEGF (1 : 500, sc-7269) and *β*-Catenin (1 : 600, sc-7963), both from Santa Cruz Biotechnology (Santa Cruz, CA, USA) and *β*-actin (1 : 1000, A2228, Sigma-Aldrich, St. Louis, MO, USA) and goat monoclonal antibody against occludin (1 : 500, sc-8144, Santa Cruz Biotechnology, Santa Cruz, CA, USA). Bands were visualized using chemiluminescence reagent (Thermo Scientific, Waltham, MA, USA). For quantification, the density of pixels of each band was determined by the NIH Image J 1.45s software (available at http://rsb.info.nih.gov/nih-image; developed by Wayne Rasband, NIH, Bethesda, MD). For each protein investigated the results were confirmed in three sets of experiments and data were normalized using the respective loading controls. Values were normalized to the corresponding value for *β*-actin internal control and expressed as a ratio.

### 2.7. RNA Isolation and Real-Time Quantitative Reverse Transcription-PCR (qPCR)

Total RNA was isolated from the cerebellum of each group (*n* = 4/time interval, *n* = 12 control, and *n* = 12 PNV treated per age) using Trizol reagent (Life Technologies, Gaithersburg, MD, USA). Primers used in this study and their respective assay identification numbers in the Applied Biosystem catalogue were VEGF: Rn01511601_m1, Flt-1: Rn00570815_m1, Flk-1: Rn00564986_m1. The levels of VEGF, Flt-1, and Flk-1 mRNAs were calculated relative to amplicon-specific standard curves by qPCR using 50 ng total RNA in triplicate and analyzed on an ABI Prism 7500 sequence detector, using a TaqMan Universal Master Mix. The optimal concentrations of cDNA and primers, as well as the maximum efficiency of amplification, were obtained by five-point, twofold dilution curve analysis for each gene. Each PCR contained 3.0 ng of reverse-transcribed RNA, 200 nM of each specific primer, SYBR SAFE PCR master mix, and RNAse-free water to a final volume of 20 *μ*L. All samples were run in triplicate with water as a no-template control and GAPDH as an endogenous control. Real-time data were analyzed using the Sequence Detection System 1.7 (Applied Biosystems).

### 2.8. Statistics

Data were assessed by a three-way ANOVA to compare the variables: treatment (saline treated and PNV treated), age (P14 and 8–10 weeks), and time (2, 5, and 24 hours). Groups' comparison was done using unpaired Student's* t-*test. The results were expressed as the mean ± SEM. Values of *P* ≤ 0.05 indicate significance.

## 3. Results

Animals of both ages injected with saline solution were alive and exhibited no sign of discomfort. Animals injected with PNV showed neurotoxic manifestation as described elsewhere [5]. P14 rat excitotoxic effects were more precocious than in adult rats, but the recovery was delayed relative to adults. Nevertheless, signs of recovery of intoxication started 5 hours after envenoming; one adult and one neonate rat died.

### 3.1. Blood-Brain Barrier Permeability

**Figure 1:**
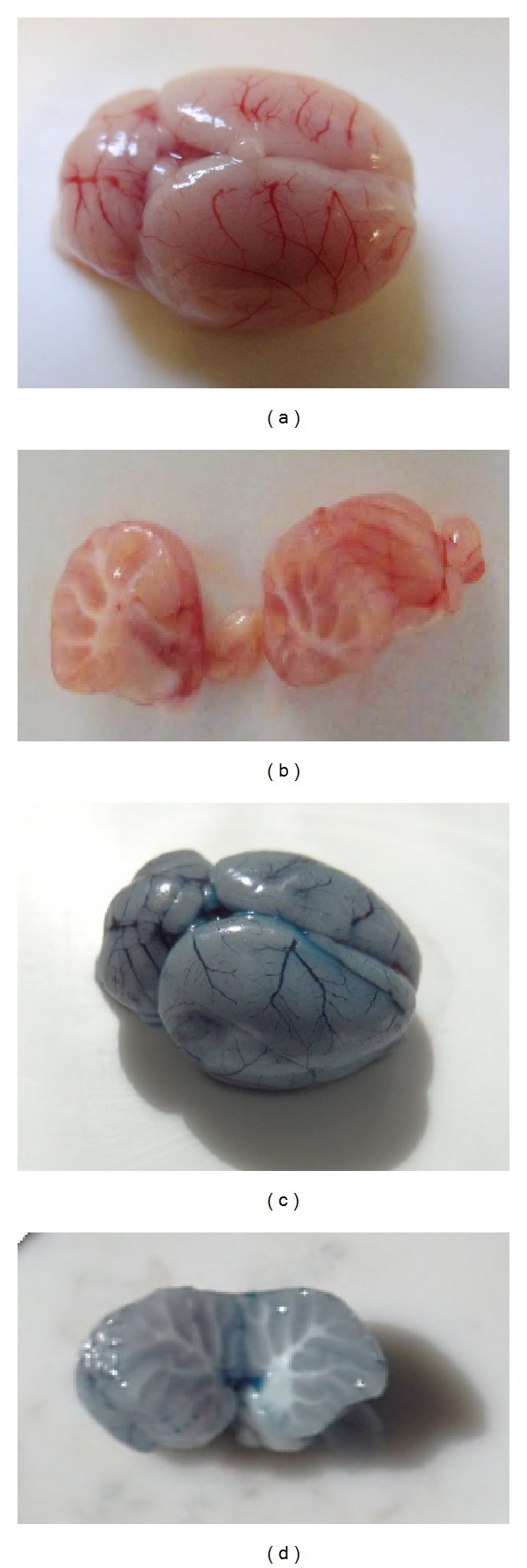
Representative photographs of brain and cerebellum from adult rats injected with Evans blue 2 hours after administration of saline ((a) and (b)) or* Phoneutria nigriventer* venom ((c) and (d)). (a) and (c) show dorsal brain surface. (b) and (d) show interior of cerebellum hemispheres.

Control rats (saline injected) that received i.v. infusion of Evans blue showed absence of any blue color on the brain surface or in the interior of cerebellum hemispheres which indicaes that there is no extravasation of Evans blue dye from microvasculature circulation (Figures 1(a) and 1(b)). On the contrary, PNV-treated rats showed brain and cerebellum surface and cerebellum interior shaded blue which indicates disruption of microvascular blood bed and leakage of Evans blue (Figures 1(c) and 1(d)). The leakage of Evans blue was detected at 2 hours, but not at 5 and 24 hours suggesting BBB return to integrity (not shown).

### 3.2. BBB Assessment: PNV Decreased Transiently Occludin, *β*-Catenin, and Laminin

**Figure 2:**
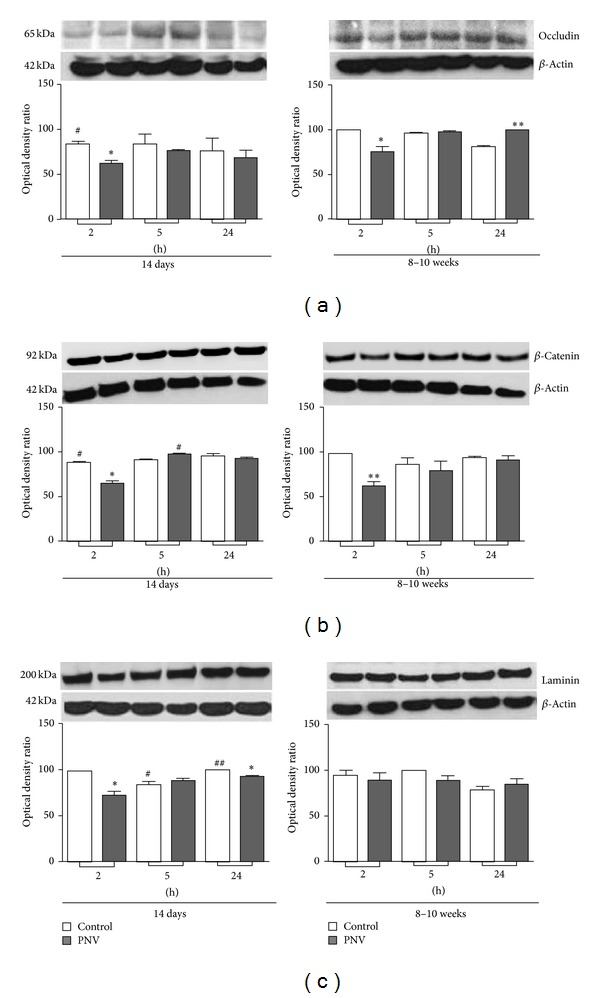
Immunoblots of occludin (a), *β*-Catenin (b), and laminin (c) show lower expression of the proteins in the cerebella lysates of envenomed animals relative to controls. Data are means ± SEM; *P ≤ 0.05; **P ≤ 0.01  ***P ≤ 0.001 relative to controls; ^#^ *P* ≤ 0.05; ^##^ *P* ≤ 0.01 relative to corresponding adults at the same time interval. Unpaired Student* t*-test.

All the three BBB-associated proteins, occludin from tight junction, *β*-catenin from adhesion junction, and laminin from the endothelial basal membrane, were decreased significantly by 34%, 35%, and 36%, respectively, 2 hours after administration of PNV to neonates. In adult rats, PNV induced a 33% decrease of occludin at 2 hours followed by 23% increase at 24 hours and 60% decrease of *β*-catenin at 2 hours and did not alter laminin expression (Figures 2(a) to 2(c)). The changes were transitory in animals of both ages.

Age-related changes showed occludin and *β*-catenin basal level lower in saline-injected neonate rats at 2 hours relative to adult rats. Moreover, baseline expression of laminin was lower in neonates at 5 hours and higher at 24 hours compared to adults. In envenomed rats, occludin was higher in adult rats at 5 hours than in P14 rats.

**Figure 3:**
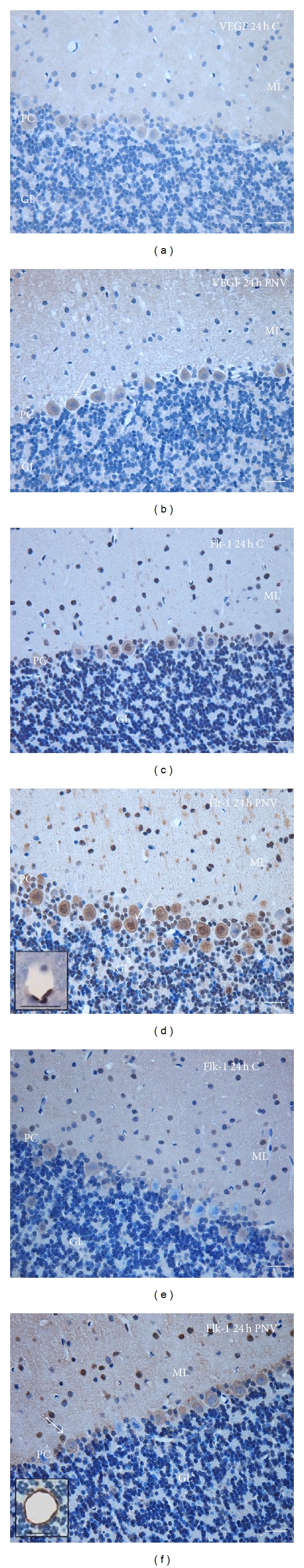
Photomicrographs of cerebellar cortex of rats aged 14 days 5 hours after administration of saline solution ((a), (c), and (e)) or* Phoneutria nigriventer* venom (PNV) ((b), (d), and (f)). PNV increased VEGF (b), Flt-1 (d), and Flk-1 (f) labeling. ML = molecular layer; PC = Purkinje cells, and GL = granular layer. Arrows point Purkinje cells positive for VEGF, Flt-1, and Flk-1; triangles show nonuniformly scattered labeled nuclei inside the molecular layer. Bars = 25 *μ*m for all panels. Inserts illustrate the expression of Flt-1 receptor in the endothelium nuclei, whereas Flk-1 receptor is expressed in nuclei and cytoplasm of endothelial cells of envenomed rats (Bars = 10 *μ*m (d) and 25 *μ*m (f)).

*Immunohistochemistry: PNV Increased the VEGF, Flt-1, and Flk-1 Reactivity*. In P14 and adult controls, VEGF immunolabeling was weakly detected in the perikaryum, outlining the cell profile of Purkinje cells (PCs); the nucleus was negative. Some cells in the molecular (ML) and granular layer (GL) were stained (Figure 3(a)). PNV-administered animals induced anti-VEGF staining in the nucleus and increased it in the somata and marginal cytoplasm of PCs (Figure 3(b)). In addition, delicate VEGF+ tangled cell processes were labeled within the ML. Cells in the GL and ML remained VEGF negative. The labeling pattern was fairly similar throughout time; however, the intensity of the reaction was marginally strongest at 24 hours.

In the cerebellum of controls, the anti-Flt-1 and anti-Flk-1 reactivity was seen in the PC nucleus and perikaryum or only in the peripheral perikaryum; a number of stained nuclei were labeled within the ML and GL (Figures 3(c) and 3(e)). PNV upregulated Flt-1 and Flk-1 level at all times in cells throughout the cerebellar cortex such as in the PCs' nuclei, somata, and peripheral cytoplasm, in nuclei within the GL and ML, and in straight-lined processes present in the ML; in envenomed rats, regions of glomeruli were wider and density of glomerular neurons seemed lower than in controls in some parts of the GL (Figure 3(d)). Flk-1 upregulation was not as intense as for Flt-1 (compare Figures 3(e) and 3(f)). Flt-1 and Flk-1 staining increased with time, but visually no obvious difference in the intensity of the labeling could be perceived between P14 and adults (not shown).

### 3.3. Western Blot and Real-Time PCR

VEGF, Flt-1, and Flk-1 expressions were higher in neonates than in adult animals, both basally and after envenomation. PNV altered level of the proteins and respective mRNAs but the dynamics of changes differed between neonates and adults.

**Figure 4:**
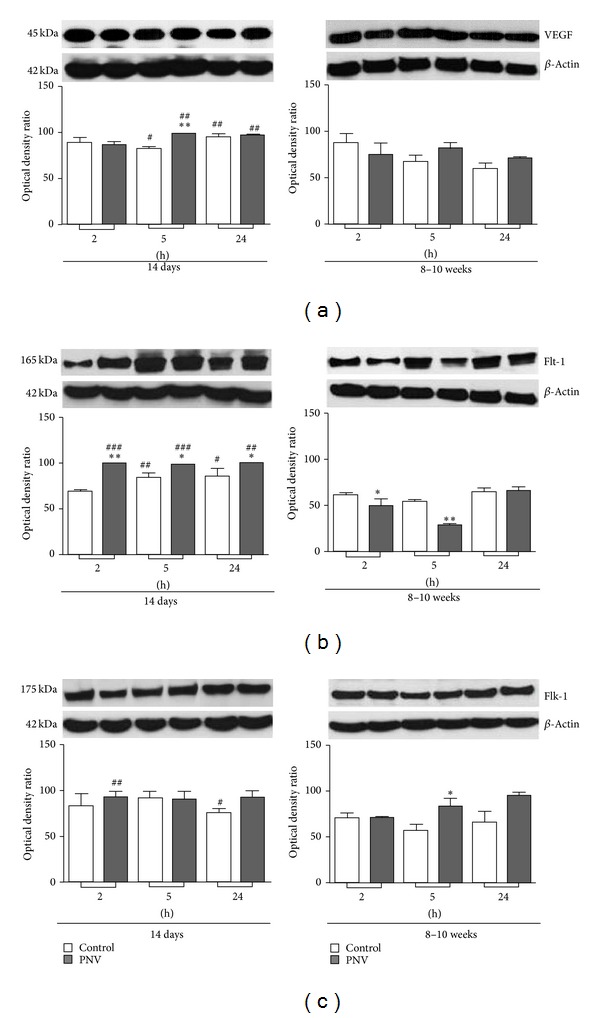
VEGF (a), Flt-1 (b), and Flk-1 (c) graph representation of western blot signals after densitometric measurement and normalization to internal *β*-actin at time points after PNV (1.7 mg/kg) or 0.9% saline i.p. injection. Data are means ± SEM. *P ≤ 0.05 and **P ≤ 0.01 in relation to controls; ^#^ *P* ≤ 0.05, ^##^ *P* ≤ 0.01, and ^###^ *P* ≤ 0.001 in relation to corresponding adults at the same time interval. Unpaired Student* t*-test was used.

**Figure 5:**
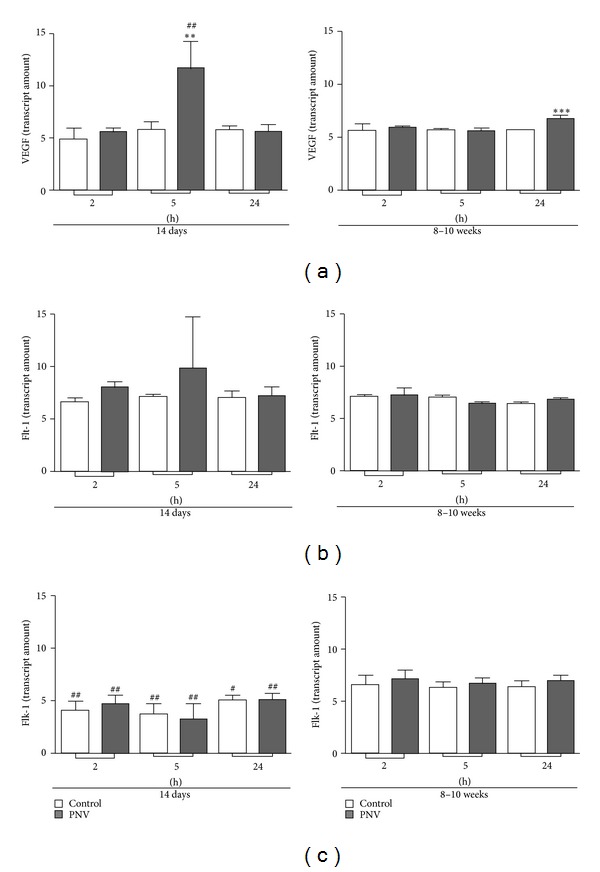
Quantitative real-time PCR analysis quantified and normalized to endogen control (GAPDH). VEGF (a), Flt-1 (b), and Flk-1 (c) mRNAs expression at time points after PNV (1.7 mg/kg) or 0.9% saline i.p. injection. **P ≤ 0.01 and ***P ≤ 0.001 indicate significant difference relative to controls; ^#^ *P* ≤ 0.05 and ^##^ *P* ≤ 0.01 denote significant age-related differences between PNV-treated or control group at corresponding time point. Student* t-*test; data were shown as means ± SEM.

Throughout the time interval examined, VEGF, Flt-1, and Flk-1 expressions were significantly higher in neonates than in adults, both controls and envenomed (Figures 4(a) to 4(c)). However, Flk-1 mRNA was higher in adults, both controls and envenomed, than in neonates at all three time points (Figure 5(c)).

PNV induced an 18% increase of VEGF expression in P14 animals at 5 hours (Figure 4(a)). VEGF mRNA content was quite similar in control and envenomed neonates and adults (Figure 5(a)), except at 5 and 24 hours when there was a twofold increase for neonates and an 18% increase for adults.

In relation to VEGF receptors, PNV caused an immediate 50% increase in the Flt-1 level in P14 rats (2 hours) that changed to 14% increase at 5 hours and 11% increase at 24 hours above baseline (Figure 4(b)). In contrast, in adult rats PNV promoted decreases in the level of Flt-1, 25% at 2 hours, and 93% at 5 hours followed by recovery to baseline values at 24 hours. In neonate and adult rats, Flt-1 mRNA expression was unaffected by PNV exposure (Figure 5(b)). PNV did not alter the expression of the Flk-1 and its mRNA in neonates (Figures 4(c) and 5(c)) but increased Flk-1 expression in adults at 5 hours.

The three-way analysis of variance showed that variables treatment versus age versus time influenced VEGF mRNA expression (*P ≤ 0.05) and Flt-1 receptor expression (*P ≤ 0.05). In addition, there was interaction between treatment versus age for the Flt-1 receptor (***P ≤ 0.001) and treatment versus time for VEGF (*P ≤ 0.05).

PNV increased CaB in neonate and adult rats while GAD was increased only in neonate rats.

**Figure 6:**
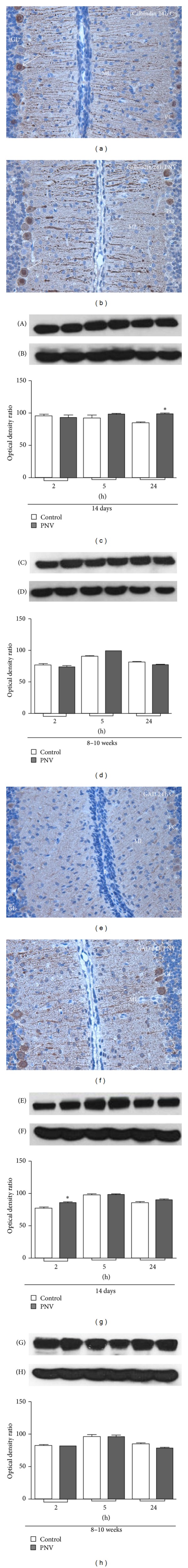
CaB and GAD immunohistochemistry in the cerebellar cortex of rats aged 14 days after i.p. injection of saline solution ((a) and (e)) or* P. nigriventer* venom PNV ((b) and (f)). (a) and (b) show CaB labeling (24 hours after saline or PNV exposure, resp.) in Purkinje cell bodies, including nuclei (arrows) and processes crossing the molecular layer (ML). (c) and (d) show immunoblots and representative histograms of the densitometric CaB values of rats injected with saline (A) or PNV (C) at different time points. (e) and (f) show GAD labeling in Purkinje cell bodies and cell processes within the molecular layer (arrow). The physiologic GAD labeling was weak in controls (e) whereas it was strong in envenomed animals (f). (g) and (h) show immunoblots and representative histograms of the densitometric GAD values of rats injected with saline (E) or PNV (G) at different time points (*n* = 6/time). The membranes were stripped and reprobed to *β*-actin, confirming equal protein loading in the gel ((B), (D), (F), and (H)). Values are mean ± SEM; unpaired Student* t*-test. *P ≤ 0.05; **P ≤ 0.01; ***P ≤ 0.001 compared to control at each time point; ^#^ *P* ≤ 0.05; ^##^ *P* ≤ 0.01; ^###^ *P* ≤ 0.001 compared to corresponding adults at the same time interval. P = pia mater; PC = Purkinje cells; ML = molecular layer; GL = granular layer. Bars = 25 *μ*m for all panels.

In controls, adult and neonate, the Purkinje cell-specific calcium-binding protein, calbindin-D28k, was expressed in the nucleus, cytosol, and dendritic tree of PCs (Figure 6(a)). Following PNV injection anti-CaB labeling was stronger particularly in PC dendrites extending across the ML (Figure 6(b)). Western blot analyses confirmed that PNV induced a 16% significant upregulation of the protein at 24 hours in neonates and a 9% increase at 5 hours in adult rats (Figure 6(c)). The baseline expression of CaB was significantly higher in neonates than in adults at 2 hours (23%); also the PNV-induced CaB expression was higher in neonates than in adults at 2 hours (25%) and 24 hours (28%). The three-way analysis of variance showed that the variables treatment versus age versus time influenced CaB expression (**P ≤ 0.01).

GABA signaling was altered in PNV-administered rats, given the expression of GAD, responsible for GABA synthesis, and was noticeably increased in PCs somata and dendritic tree (Figures 6(e) and 6(f)). Immunoblots showed that the GAD protein expression of treated P14 rats was 10% upregulated at 2 hours and remained practically unchanged thereafter (Figure 6(g)). In adult rats exposed to PNV, GAD remained unchanged compared to baselines values (Figure 6(h)). There was an age-related difference at 24 hours with GAD expression of PNV-treated neonate rats surpassing by 15% that of PNV-treated adults. The three-way analysis of variance showed interaction only between treatment versus age (***P ≤ 0.001).

## 4. Discussion

The present findings show that prominent increases of VEGF and receptor Flt-1 in cerebellar neurons course with neurotoxic effects caused by PNV in P14 rats. This is accompanied by upregulations of VEGF mRNA, CaB, and GAD. The data suggest that, upon systemic presence of PNV, endogenous signaling mechanisms may have activated transcription factors and promote proteins translational regulation in newborn rats.

The alterations involving the VEGF-Flt-1 binding in neonate rats were concurrent with prominent decreases of the proteins associated with BBB endothelium: occludin, *β*-catenin, and laminin. The data corroborate previous studies showing PNV-induced BBB permeation in adult rats [3–6], now reaffirmed by the extravasation of Evans blue injected peripherally. Herein, the time course data over a period of 2 hours to 24 hours revealed that expressional decreases of occludin, *β*-catenin, and laminin (at 2 hours) were simultaneous with the peak of Flt-1 upregulation (50%) but preceded the PNV-induced significant increases of VEGF and VEGF mRNA, which occurred later at 5 hours after PNV. Also, at 5 hours, occludin, *β*-catenin, and laminin levels were recovered markedly relative to corresponding control levels, which paralleled with content of VEGF and Flt-1 significantly above baseline. Such data disclose what seems to be a coordinated sequence of molecular events, which began with the BBB breakdown, and proceeded with increases of Flt-1, VEGF, and VEGF mRNA. The transient disturbance of BBB-associated proteins in P14 rats might be due to the fact that VEGF receptors once activated trigger signals directed to endothelial cells [18] and neuron cell [19], either to accentuate permeability [20–22] and/or to restore homeostasis.

Curiously, in contrast to neonates, PNV induced decreases over time of Flt-1 in adult rats reaching ~93% downregulation at 5 hours, which though was transient and shows great capacity for recovery since at 24 hours the baseline level had been already reached. Consistent with this, at the same 24 hours, VEGF mRNA was significantly increased. However, PNV increased cerebellar Flk-1 and CaB at 5 hours, a time point in which BBB functionality had been restored as indicated by absence of extravasation of Evans blue dye. Interestingly, 5-hour time coincides with the beginning of the adult animals' recovery from neurotoxic manifestations caused by PNV [4, 6].

VEGF, Flt-1, and Flk-1 protein expressions were also detected by immunohistochemistry. The three proteins were constitutively present in Purkinje neurons and their dendritic processes extended across the ML in control animals. The exposure to PNV induced visible increases in the Purkinje neurons' immunoreactivity over a period of 2 to 24 hours; interestingly, nonuniformly dispersed cells within the GL and ML also showed increases in the Flt-1 and Flk-1 reactivity in envenomed P14 and adult rats. We do not know whether such cells are astrocytes and/or neurons. Studies have shown that VEGF and Flk-1 can be expressed by granule neurons during postnatal development of cerebellum [23–25] and by astrocytes under normal or pathological conditions [26–29]. Astrocytes also express Flt-1, but as far as we know there is no mention in the literature on Flt-1 expression by cerebellar granule cells. Since the ML is characterized by extensive synaptic contacts between granule neurons, interneurons, Begmann glia, and Purkinje neurons [30], it is conceivable that activation of a given cell may elicit a chain of stimuli in the others. The induction of VEGF and receptors in neurons and probably in astrocytes supports previous evidence showing these cells as targets of PNV concurrently with BBB dysfunction [3–9, 17].

VEGF family members, traditionally known as potent inducers of angiogenesis, have been recently recognized to exert various nonangiogenic effects on different cell types, among which there are neuron cells [13]. VEGF and receptors have been recognized as an important element for neuron survival and maintenance of endothelium in adults. It acts in the nervous system both through vascular and neuronal mechanisms. Molecules that dually affect both neural and vascular (neurovascular) functions are referred to as angioneurins; their action includes regulation of angiogenesis, BBB integrity, vascular perfusion, neuroprotection, neurodegeneration, and synaptic plasticity [31]. The protection of neurons by VEGF is exerted by interference on Ca^2+^, K^+^, and voltage-gated Na^+^ channels physiology and modulation of glutamatergic synaptic excitability [11–13]. VEGF inhibits outward delayed K^+^ currents and reduces Ca^2+^ influx through the high-voltage-activated Ca^2+^ channels; the cytokine also inhibits Na^+^ currents in cultured rat hippocampal neurons, thus modulating neuron excitability. In hippocampus, the cytokine is considered an endogenous anticonvulsing factor to preserve pyramidal neurons from hyperexcitability [14]. The interference of PNV peptides in calcium homeostasis and glutamate handling have been well-documented [32–36]. Herein, we found signs that PNV disturbed calcium buffering in Purkinje neurons. P14 and adult rats treated with PNV increased CaB immunoreactivity in the PC's nucleus, perikaryum, and dendritic ramification, which was confirmed by the WB data showing upregulation of the protein. Because PCs are the only output cell of the cerebellar cortex and because CaB functions as a calcium buffer, the changes in the PC's immunoreactivity and level of the protein in the cerebellum lysates suggests implication on PC functional changes related with PNV effect.

PNV induced an immediate, but transient, increase of GAD expression in neonate rats, while in adults it was unaffected. The key synthesizing isoforms for GABA, GAD types 65 and 67, were decreased in the Purkinje cells of neonate rats but not in adult rats treated with PNV. This suggests that the decarboxylation of glutamate to the major inhibitory neurotransmitter GABA supports the view that synaptic inhibition is vital for the control of neuron excitability in the central nervous system and a dynamic mechanism to restore brain homeostasis, when disturbed by neurotoxic peptides of PNV. The increase of GAD in neonate rats in response to PNV is in accordance with the major level of VEGF both basically and after PNV treatment in neonate than in adult rats (see Figure 4). Whether the increase of VEGF promoted by PNV, mainly seen in neonate rats, was a way to preserve neurons [11, 12] against the glutamate toxicity generated by PNV is uncertain. Nevertheless, it is well-known that the postnatal development of cerebellum continues during months after birth [37], which requires active angiogenesis, neurogenesis, and cell migration. These cell processes are regulated by angioneurins, that is, growth factors that act both in neural an vascular cells, like VEGF and receptors [10, 13, 19]. In contrast, angiogenesis, neurogenesis, and cell migration are very discrete or switched off in adult animals. This might explain the relative lower expression of VEGF and receptors of cerebellum in adult animals, what could imply a mature BBB able to better stand the toxic effects. In addition, the BBB in neonate rats is undergoing postnatal development [38], what could explain why junctional proteins remains below baseline at 24 h in P14 rats whereas it is above baseline in adult rats. It could be the reason why systemic PNV increases even more the expression of VEGF and its mRNA (at 5 hours) and the receptors Flt-1 (from 2 to 24 hours) for neonates and transiently decreases Flt-1 expression but does not alter VEGF and only increases VEGF mRNA at 24 h in adults. Interestingly, PNV causes release of kallikrein [39], and kallikrein causes BBB breakdown [40]. VEGF and kallikrein share a series of effects such as enhancing the survival and migration of neuronal and glial cells, promoting angiogenesis, protecting against ischemia, apoptosis, and glutamate-induced neurotoxicity [41]. However, we do not know if kallikrein released by PNV is able to increase VEGF.

## 5. Conclusions

VEGF and PNV have in common to act on BBB-enhanced permeability, interfere in ion channels physiology, affect synaptic plasticity, and disturb glutamatergic transmission. Here, we found that the evolution of the toxic manifestations exhibited by rats injected with PNV seems to be time-related to the dynamics of immunochemical content of VEGF, Flt-1, and Flk-1 and respective mRNAs and CaB and GAD in the cerebellum. Age-related differences with neonate rats apparently were also found more susceptible to PNV than adult rats. Future studies are needed to determine whether VEGF and tyrosine kinase intracellular domains changes in Purkinje cells underlie BBB disturbances as a consequence of venom effect or a causal factor mediating the venom's homeostatic perturbations. Ionic and glutamate disturbances, induced by PNV, affect synaptic extracellular compartments and neuronal signaling and could underlie the neurotoxic manifestations of animals. Whether the increase of VEGF in neurons represents protective modulation of synaptic excitability and of Ca^2+^, K^+^, and Na^+^ channels functioning is a matter to be elucidated.
